# Supplementary material for: Identification of anticancer drugs for hepatocellular carcinoma through personalized genome‐scale metabolic modeling
Source: Mol Syst Biol. 2014 Mar 28;10(3):721. doi: 10.1002/msb.145122 (PMC4017677; doi:10.1002/msb.145122)
Supplement: Supplementary file 1 — Supplementary Figure S1 [file MSB-10-3-721-s37.pdf]

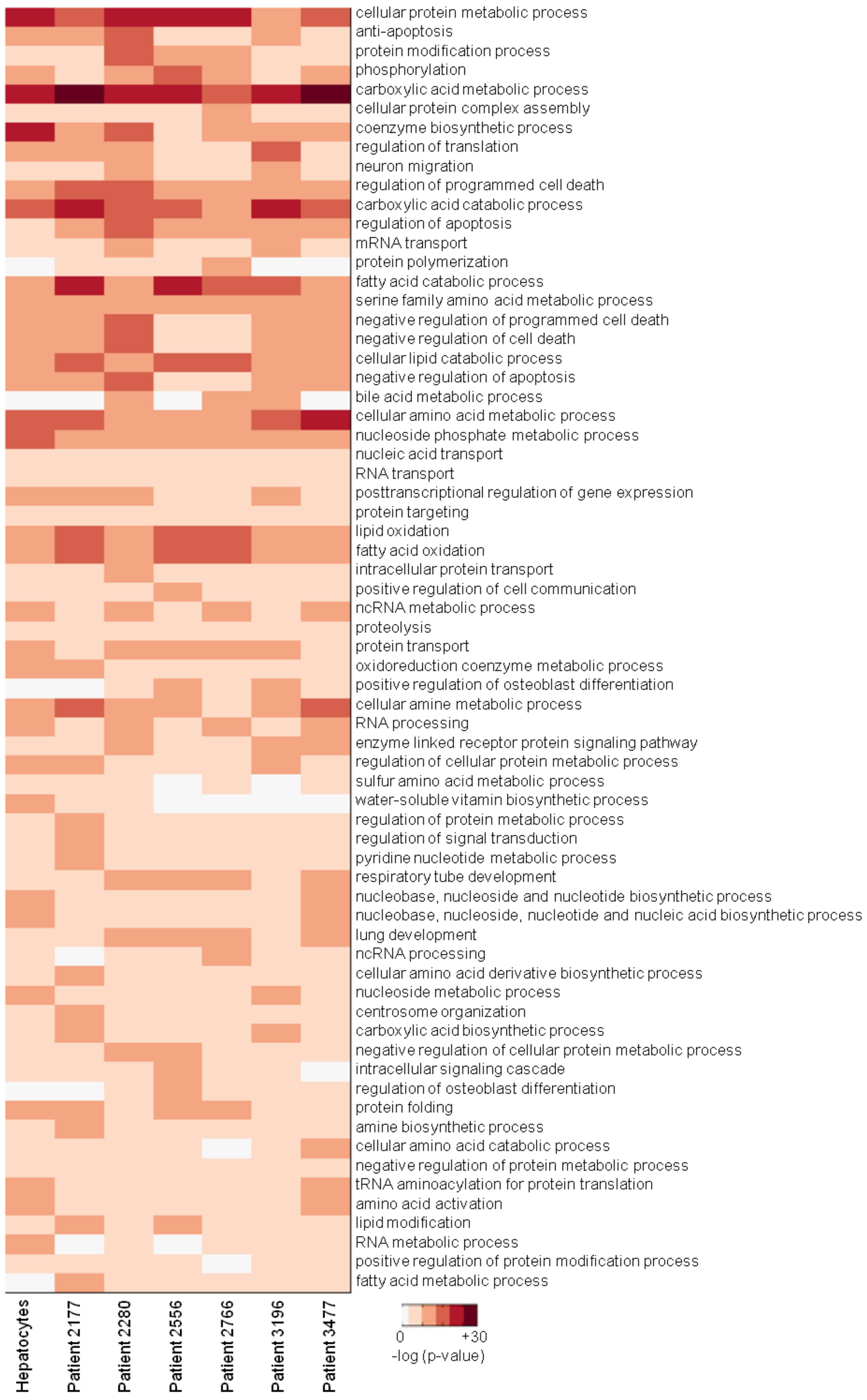

Figure S1 Functional differences between the measured 4,936 proteins in all six HCC patients and hepatocytes are presented based on the level 5 gene ontology biological process (GO BP) terms (p-value<0.005).
