# Supplementary material for: Identification of anticancer drugs for hepatocellular carcinoma through personalized genome‐scale metabolic modeling
Source: Mol Syst Biol. 2014 Mar 28;10(3):721. doi: 10.1002/msb.145122 (PMC4017677; doi:10.1002/msb.145122)
Supplement: Supplementary file 2 — Supplementary Figure S2 [file MSB-10-3-721-s39.pdf]

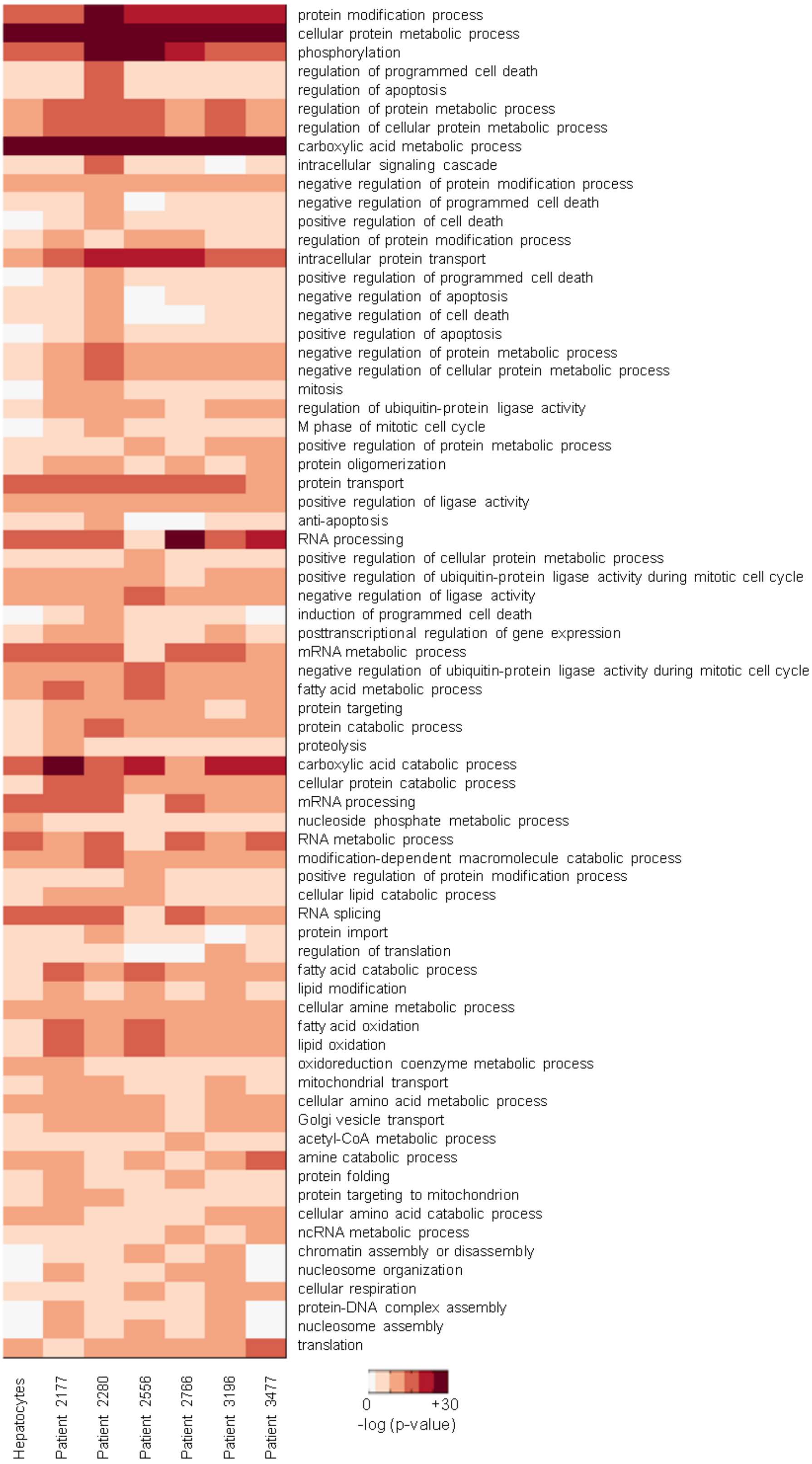

Figure S2 The average protein expression data of 27 HCC patients were used where a protein hadn't been measured in all six patients during the reconstruction of the personalized GEMs. Functional differences between the 15,841 proteins in hepatocytes and six HCC patients are presented based on the level 5 gene ontology biological process (GO BP) terms (p-value<0.0001).
