# Supplementary material for: Identification of anticancer drugs for hepatocellular carcinoma through personalized genome‐scale metabolic modeling
Source: Mol Syst Biol. 2014 Mar 28;10(3):721. doi: 10.1002/msb.145122 (PMC4017677; doi:10.1002/msb.145122)
Supplement: Supplementary file 3 — Supplementary Figure S3 [file MSB-10-3-721-s41.pdf]

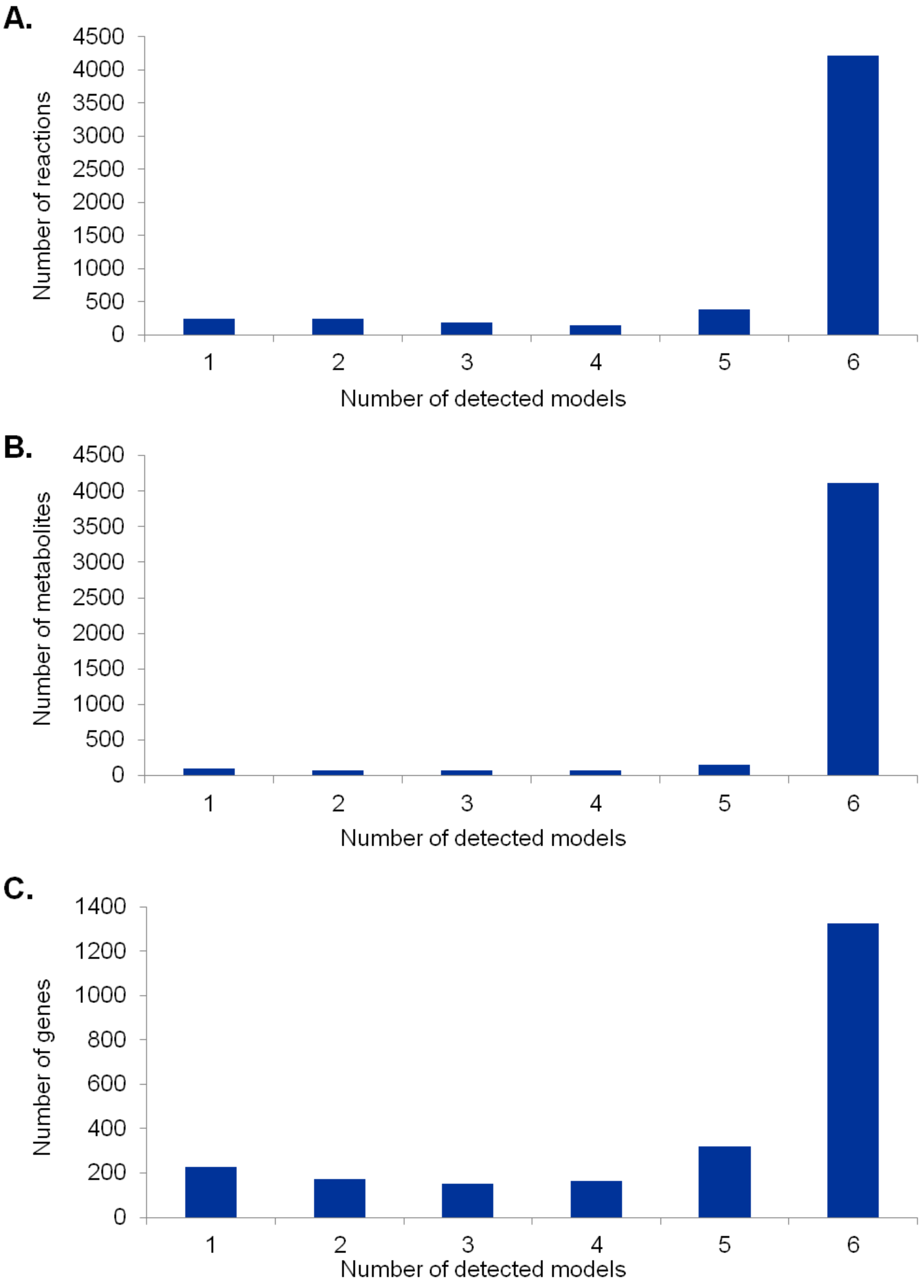

Figure S3 The number of the reactions (A), metabolites (B) and genes (C) detected in the personalized models.
